# ARID1B Deficiency Leads to Impaired DNA Damage Response and Activated cGAS-STING Pathway in Non-Small Cell Lung Cancer

**DOI:** 10.7150/jca.91955

**Published:** 2024-03-11

**Authors:** Guangsheng Zhu, Jinghao Liu, Yongwen Li, Hua Huang, Chen Chen, Di Wu, Peijun Cao, Lianchun Su, Yanan Wang, Hongbing Zhang, Hongyu Liu, Jun Chen

**Affiliations:** 1 Department of Lung Cancer Surgery, Tianjin Medical University General Hospital, Tianjin 300052, People's Republic of China.; 2 Tianjin Lung Cancer Institute, Tianjin Key Laboratory of Lung Cancer Metastasis and Tumor Microenvironment, Tianjin Medical University General Hospital, Tianjin 300052, People's Republic of China.

**Keywords:** ARID1B mutation, NSCLC, ICIs, cGAS-STING pathway, Chromatin Accessibility, DNA Damage

## Abstract

**Purpose:** Lung cancer is a major cause of morbidity and mortality globally, necessitating the identification of predictive markers for effective immunotherapy. Mutations in SWI/SNF chromatin remodeling complex genes were reported sensitized human tumors to immune checkpoint inhibitors (ICIs), but the underlying mechanisms are unclear. This study aims to investigate the association between SWI/SNF gene ARID1B mutation and ICI response in non-small cell lung cancer (NSCLC) patients, to explore the functional consequences of ARID1B mutation on DNA damage response, immune microenvironment, and cGAS-STING pathway activation.

**Methods:** TCGA LUAD, LUSC, and AACR GENIE data are analyzed to assess ARID1B mutation status in NSCLC patients. Prognostic analysis evaluates the effect of ARID1B mutation on patient outcomes. In vitro experiments carried to investigate the consequences of ARID1B knockdown on DNA damage response and repair. The immune microenvironment is assessed based on ARID1B expression, and the relationship between ARID1B and the cGAS-STING pathway is explored.

**Results:** ARID1B mutation frequency is 5.7% in TCGA databases and 4.4% in the AACR GENIE project. NSCLC patients with ARID1B mutation showed improved overall and progression-free survival following ICIs treatment. ARID1B knockdown in lung cancer cell lines enhances DNA damage, impairs DNA repair, alters chromatin accessibility, and activates the cGAS-STING pathway. ARID1B deficiency is associated with immune suppression, indicated by reduced immune scores, decreased immune cell infiltration, and negative correlations with immune-related cell types and functions.

**Conclusion:** ARID1B mutation may predict improved response to ICIs in NSCLC patients. ARID1B mutation leads to impaired DNA damage response and repair, altered chromatin accessibility, and cGAS-STING pathway activation. These findings provide insights into ARID1B's biology and therapeutic implications in lung cancer, highlighting its potential as a target for precision medicine and immunotherapy. Further validation and clinical studies are warranted.

## Introduction

Lung cancer remains a leading cause of morbidity and mortality worldwide, with over 2.2 million new cases and 1.8 million deaths annually 1. Non-small cell lung cancer (NSCLC), including adenocarcinoma (LUAC), squamous cell carcinoma (LUSC), and large cell carcinoma, accounts for 80-85% of all lung cancer cases 1,2. Despite advancements in NSCLC treatment, the overall five-year survival rate remains below 30% 1,2. Immune checkpoint inhibitors (ICIs), such as anti-PD-1 and anti-PD-L1 antibodies, have revolutionized cancer immunotherapy but show a limited overall response rate of 20-30% 3,4.

PD-L1 expression on tumor cells is commonly used as a biomarker to predict response to ICIs 5. Higher PD-L1 expression is associated with immune activation and increased response to anti-PD-1 and anti-PD-L1 therapies 6. However, PD-L1-negative tumors can still respond to anti-PD-1 therapy, and only a fraction of patients with high PD-L1 expression show response, suggesting additional factors determine ICI response 3,4. Tumor mutational burden (TMB) is another recognized biomarker associated with response to ICIs, as higher mutational loads lead to increased tumor visibility and a more potent antitumor T cell response following ICI treatment 5.

Recent studies, including our own, have identified mutations in genes of the SWI/SNF chromatin remodeling complex that sensitize human tumors to ICIs 7-9. Patients with mutations in ARID1A, ARID1B, and ARID2 of the SWI/SNF complex exhibit significantly higher TMB and neoantigen loads 8. Furthermore, these mutations alter the immune microenvironment, including decreased activated dendritic cells and monocyte infiltration 8. However, the role of the epigenetic landscape of tumor cell chromatin in ICI sensitivity and the mechanism underlying SWI/SNF complex mutations in T cell activity remain poorly understood.

The SWI/SNF chromatin remodeling complex, crucial for chromatin stability, gene expression, and post-translational modifications, frequently harbors mutations in various cancers 7,8. ARID1B and its paralog ARID1A encode the largest subunits of the complex and play key roles in cell fate decisions 7. ARID1B mutations, including protein loss resulting from nonsense, frameshift, and insertion/deletion mutations, are prevalent in human diseases and cancers 7. Epigenetic and transcriptional regulation, along with post-translational modifications, contribute to downregulation of ARID1B in tumors with low mutational rates 7. ARID1B is involved in fundamental cellular processes such as transcriptional regulation, DNA replication, DNA damage repair, genomic stability, apoptosis, cell proliferation, and cell differentiation.

In this study, we investigate the impact of ARID1B function loss on DNA damage and immune microenvironment changes in NSCLC. Our findings shed light on the interplay between these factors and their potential implications for immune modulation. Leveraging ARID1B-mediated immune activation may pave the way for novel therapeutic strategies to enhance the clinical outcomes of NSCLC patients treated with ICIs.

## Materials and Methods

### Data source

We utilized data from 1,053 NSCLC patients from the TCGA LUAD and LUSC datasets 10 and DNA sequencing data from 2,004 patients from the AACR GENIE project. We obtained overall survival data and DNA sequencing results of 1661 cancer patients 11 who underwent immunotherapy from cbioportal (http://www.cbioportal.org/). Additionally, we retrieved progression-free survival data and DNA sequencing information of 350 non-small cell lung cancer (NSCLC) patients 12-14 who received immunotherapy from cbioportal.

### Cells and cell culture

H2030 and SK-mes-1 NSCLC cell lines were obtained from the ATCC and cultured in RPMI 1640 and DMEM medium, respectively, supplemented with 10% FBS. Cells were maintained at 37°C in a 5% CO2 humidified atmosphere.

### Western blot

Protein samples were extracted and quantified, and agarose gel electrophoresis was performed using 10% separating gels. Proteins were then transferred onto PVDF membranes (Millipore, Billerica, MA, United States) using a semi-dry transfer system. The membranes were then blocked with 5% skim milk at room temperature for 2 h. The primary antibody and membranes were incubated overnight at 4°C. Subsequently, the membranes and secondary antibody (1: 5000 dilution; Thermo Fisher Scientific, Inc.) were incubated for 1 h at room temperature. The bands were visualized using the Pierce ECL substrate (Thermo Fisher Scientific, Inc.).

### siRNA interference and lentivirus infection

The siRNA targeting human ARID1B was synthesized by Ribobio (Guangzhou, China) and transfected into cells using Lipofectamine 2000 (Life Technologies, New York, USA) following the manufacturer's protocol. Lentiviral vectors encoding short hairpin RNA (shRNA) against human ARID1B and control sequences were purchased from Genechem (Shanghai, China). Transfection efficiency was assessed by protein immunoblotting.

### Immunofluorescence

NSCLC cells were seeded on 12-well plates with coverslips, fixed in 4% paraformaldehyde, permeabilized with 0.5% Triton X-100, blocked with 1% BSA, and incubated with the primary antibody against γ-H2AX (diluted 1:200; Abcam, UK) overnight at 4°C. After washing, cells were incubated with the fluorescent secondary antibody, and coverslips were mounted using fluorescence quenching mounting medium with DAPI. Fluorescence images were captured using a microscope.

### Comet assay

The comet assay was performed using the abcam assay kit (Abcam, UK) following the manufacturer's protocol. Cells were diluted to 1x10^5 cells/mL and mixed with comet agarose (1:10 ratio). The mixture was applied to comet-specific slides and incubated at 4°C for 15 minutes. Slides underwent lysis, alkaline treatment, electrophoresis, and ethanol immersion. Vista Green DNA stain was added, and cells were incubated before examination under a fluorescence microscope with FITC filter.

### Micrococcus Nuclease experiment

Cells were washed with chilled PBS and lysed with 100 μL of lysis buffer on ice for 30 minutes. Digestion with a mixture containing MNase (Life Technologies, New York, USA) was performed. Proteinase K solution was added, and samples were mixed thoroughly. DNA was extracted using the TIANamp Genomic DNA Kit (TIANGEN, Beijing, China) and subjected to horizontal electrophoresis.

### T cell-mediated tumor cell killing assay

Human PBMCs were cultured in RPMI-1640 medium and activated with Dynabeads™ Human T-Activator CD3/CD28 (Gibco, CA, USA) for 1 week according to the manufacturer's instructions. NSCLC cells were seeded into 12-well plates at a cell-dependent concentration. After 24 h, activated PBMCs were cocultured with adhered NSCLC cells for 48 h at a ratio of 3:1. After 48 hours of incubation, remove the cultured and suspended cells, wash them with PBS, and perform crystal violet staining and take photos.

### Bioinformatics and statistical methods

Differential gene analysis utilized the 'edgeR' R package 15, while pathway enrichment analysis employed the 'clusterProfiler' R package 16. Immune microenvironment scoring 17 and analysis of immune cell infiltration were performed using the GSVA package 18. Immune cell infiltration results obtained from the Sangerbox website (http://vip.sangerbox.com/) included CIBERSORT 19, EPIC 20, MCPcounter 21, IPS 22, and QUANTISEQ 23. Statistical significance was defined as a p-value ≤ 0.05. **** denotes a p-value ≤ 0.0001, *** denotes a p-value ≤ 0.001, ** denotes a p-value ≤ 0.01, and * denotes a p-value ≤ 0.05.

## Results

### ARID1B mutation is associated with Chromatin damage and ICIs response in NSCLC Patients

**Figure 1:**
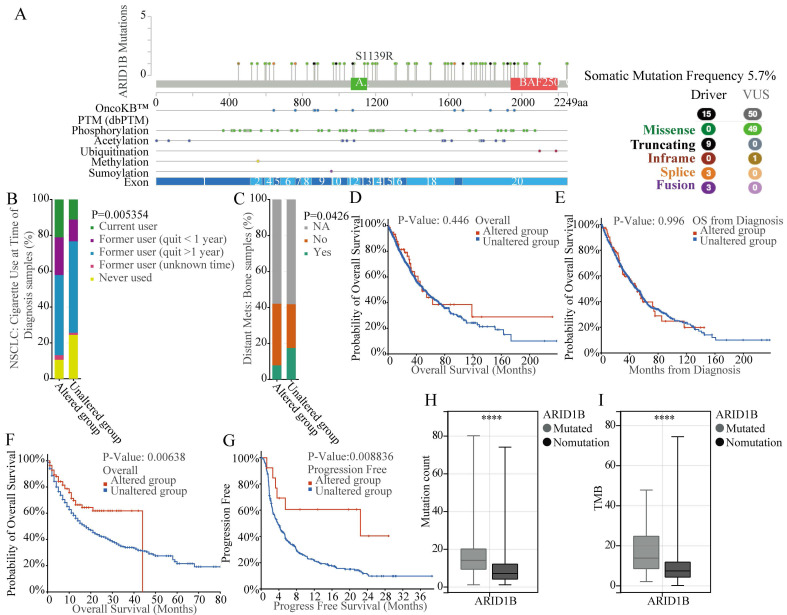
** Mutation analysis and prognostic significance of ARID1B in NSCLC.** (**A**) Mutation frequency and types, as well as predicted PTM sites of ARID1B in NSCLC patients from TCGA LUAD, LUSC datasets. The distribution of mutation types across the exons of ARID1B is shown. (**B**) Association between ARID1B mutation and bone metastasis in NSCLC patients. The bar graph represents the incidence of bone metastasis in patients with or without ARID1B mutation.** C** Association between ARID1B mutation and smoking status in NSCLC patients. (**D, E**) Prognostic analysis of ARID1B mutation in NSCLC patients who did not receive ICIs treatment. Kaplan-Meier survival curves show the overall survival and progression-free survival, respectively. (**F, G**) Prognostic analysis of ARID1B mutation in patients who underwent ICIs treatment. Kaplan-Meier survival curves demonstrate the overall survival and progression-free survival, respectively. (**H, I**) Comparison of mutation counts and tumor mutation burden (TMB) between ARID1B-altered and ARID1B non-altered NSCLC patients. The box plots represent the median, interquartile range, and outliers. *p < 0.05, **p < 0.01, ***p < 0.001, ****p < 0.0001.

According to our previous study, NSCLC patients with ARID1B mutations treated with ICIs trends had better OS compared to WT group. We therefore used both TCGA as well as AACR GENIE databases to investigate the mutation status of ARID1B gene. According to our exploration of TCGA LUAD, LUSC datasets and AACR GENIE project datasets, we identified a mutation frequency of 5.7% for ARID1B gene in NSCLC patients of TCGA databases (Fig. 1A) and 4.4%in NSCLC patients of AACR GENIE project (Supplementary Fig. 1). The mutation type including missence, inframe, splice and fusion. Also, we identified some PTM location site, including phosphorylation, acetylation, ubiquitination, methylation and sumoylation. The mutations are evenly distributed across various exons of the entire gene, without the presence of hotspot mutations (Fig. 1A). The gene mutation rates of ARID1B in these two datasets are 6% (67/1053) and 5% (78/1636), respectively. Additionally, we observed a relationship between ARID1B mutation and smoking status, with a higher prevalence of ARID1B mutation in smoking patients, which indicated the tobacco exposure may significantly impact mutations of ARID1B gene (Fig. 1B). Furthermore, we found a correlation between ARID1B alteration (any form of non-synonymous mutation in ARID1B, classifies the patient as belonging to the ARID1B-altered group) and bone metastasis in NSCLC patients, with a lower incidence of bone metastasis observed in patients with ARID1B mutation (Fig. 1C).

Analyzing the prognosis of nearly 3,000 NSCLC patients (1,053 patients from TCGA LUAD and LUSC datasets and 2,004 patients from the AACR GENIE project), we determined that ARID1B mutation was not associated with prognosis in patients who did not receive ICIs (Fig. 1D, E). However, in a subsequent prognosis analysis of 1661 cancer patients who underwent ICIs therapy, we discovered that patients with ARID1B mutation exhibited better outcomes following ICIs treatment, the median overall survival time for altered group is 44 months compared to 18 months for non-altered group (Fig. 1F). The prognosis analysis of 350 NSCLC patients who underwent ICIs therapy, we also discovered that patients with ARID1B mutation exhibited better outcomes following ICIs treatment, the median progress free survival time is 22.43 months for altered group compared to 3.77 months for non-altered group (Fig. 1G). This result suggests a predictive value for ARID1B in ICIs response in NSCLC patients. Moreover, consistent with our previous findings, mutated patients demonstrated higher mutation counts and TMB (Fig. 1H, I).

In order to explore the role of ARID1B mutations in the regulation of ICIs response, we examined the pathway enrichment analysis of differentially expressed genes between mutated and non-mutated patients in sequencing data from these patients who did not receive ICI treatment in the TCGA database. We found significant enrichment in pathways related to chromatin, DNA assembly and stability, and immune response (Supplementary Fig. 2A). GSEA enrichment analysis showed that many chromatin and DNA assembly/stability related pathways associated with ARID1B mutations were downregulated (Supplementary Fig. 2B).

### ARID1B mutation leads to impaired DNA damage response and DNA repair

To explore the role of ARID1B and DNA damage response genes, we first mined the TCGA database and found a positive correlation between ARID1B expression and genes for DNA damage response and DNA repair, such as ATM, CHEK1, CHEK2, H2AFX, KU70, and KU80 (Supplementary Fig. 3A-F).

**Figure 2:**
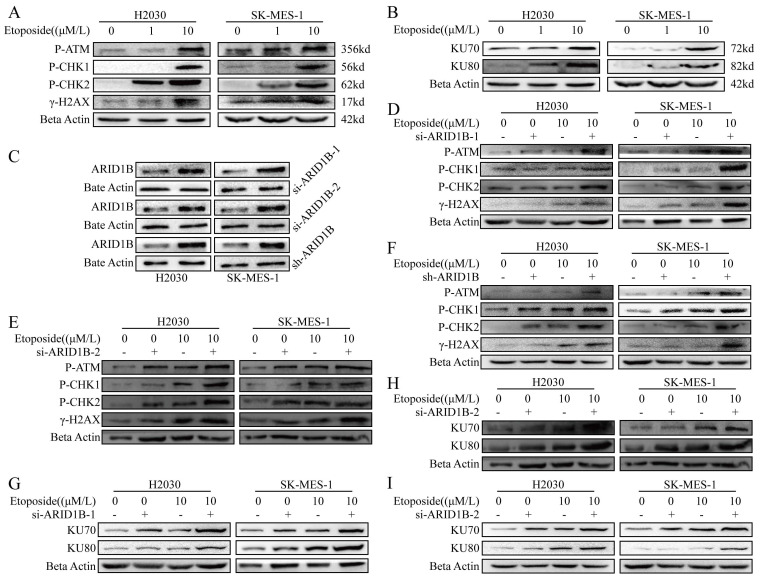
** Impact of ARID1B on DNA damage response and repair in lung cancer cells.** (**A-B**) Western blot demonstrating the levels of DNA damage response (γ-H2AX, p-CHK1, p-CHK2) and DNA damage repair (KU70, KU80) proteins in ARID1B wild-type lung cancer cells (H2030 and SK-mes-1) upon etoposide treatment. (**C**) Western blot validation of the knockdown efficiency of ARID1B by two types of siRNA and shRNA. (**D-F**) Western blot of DNA damage response proteins in ARID1B knockdown (with siRNA and shRNA) lung cancer cells (H2030 and SK-mes-1) treated with etoposide. (**G-I**) Western blot of DNA damage repair proteins in ARID1B knockdown (with siRNA and shRNA) lung cancer cells (H2030 and SK-mes-1) treated with etoposide. *p < 0.05, **p < 0.01, ***p < 0.001, ****p < 0.0001.

Next, we want to know whether ARID1B respond to DNA damage. We induced DNA damage in SK-MES1 and H2030 lung cancer cells, which are ARID1B wild type cells, with etoposide, a known DNA-damaging agent. As shown in Fig. 2, we observed DNA damage induced by etoposide in SK-MES1 and H2030 cells, as evidenced by upregulation of phosphorylated γ-H2AX, a phosphorylated histone protein, which is a marker of DNA DSBs, as well as phosphorylated CHK1, CHK2, ATM, markers of DNA damage (Fig. 2A). Since in eukaryotic organism, non-homologous end joining (NHEJ) is one of the major DNA damage repair mechanisms, we further measured the expression of KU70 and KU80, which are involved in the NHEJ pathway, by immunoblotting. The DNA damage induced by etoposide led to the upregulation of KU70 and KU80 (Fig. 2B).

In order to further elucidate the role of ARID1B in DNA damage response, as the majority of ARID1B gene mutations are loss-of-function mutations, we employed two siRNAs and shRNA to knock down ARID1B in SK-MES1 and H2030 cells. Western blot validation confirmed the high efficiency of knockdown using all three methods (Fig. 2C). Subsequently, we induced DNA damage in these cells using etoposide. We observed that si-ARID1B alone did not change the expression of the DNA damage markers, but ARID1B knockdown cells showed enhanced DNA damage when treated with etoposide, resulting in increased levels of phosphorylated CHK1, CHK2, ATM, and γH2AX (Fig. 2D,E). Similar results were obtained using lentiviral-mediated ARID1B knockdown (Fig. 2F).

Furthermore, DNA repair pathways were up-regulated in ARID1B-knockdown cells, as evidenced with increased KU70 and KU80 expression (Fig. 2G-I).

**Figure 3:**
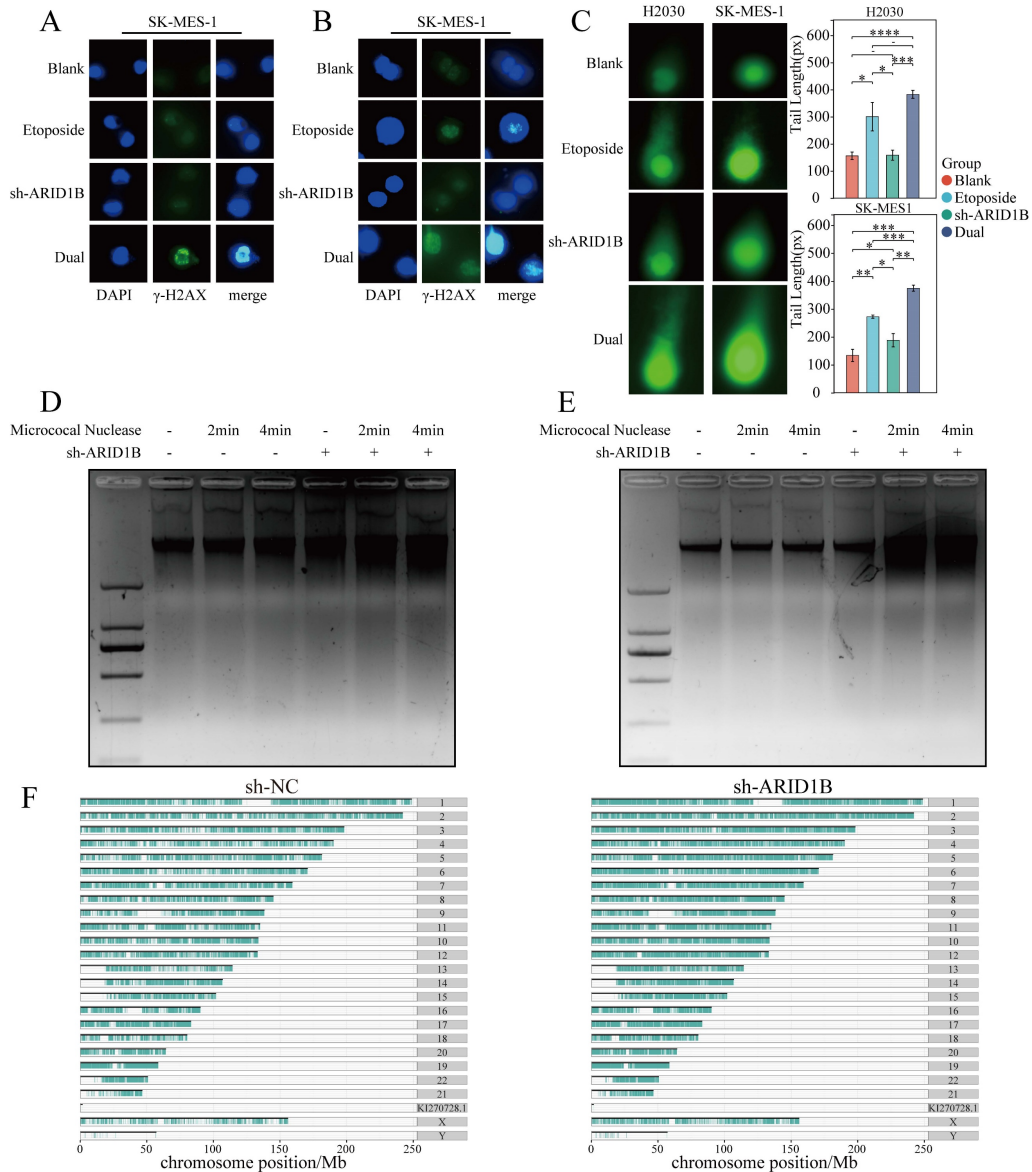
** Influence of ARID1B on chromatin accessibility and DNA damage levels.** (**A, B**) Immunofluorescence staining showing the levels of γ-H2AX in lung cancer cells with or without ARID1B knockdown upon etoposide treatment. **C** Comet assay results indicating DNA damage levels in lung cancer cells with or without ARID1B knockdown following etoposide treatment. (**D, E**) Micrococcal nuclease digestion assay demonstrating chromatin accessibility in ARID1B knockdown lung cancer cells. (F) Based on ATAC seq analysis of non-small cell lung cancer cells before and after knocking down ARID1B, plot the distribution of peaks obtained on chromosomes. The agarose gel electrophoresis images represent the DNA fragment lengths. *p < 0.05, **p < 0.01, ***p < 0.001 ****p < 0.0001.

We also confirmed the impact of loss function of ARID1B on DNA damage, with immunofluorescence experiments and the results demonstrated that SK-MES1 and H2030 cells with ARID1B gene knockdown exhibited increased upregulation of the γ-H2AX upon etoposide induction (Fig. 3A, B).

These findings suggest that ARID1B is associated with DNA damage response and modulates DNA repair pathways, highlighting its potential role in maintaining genome stability and influencing the cellular response to DNA damage.

Furthermore, comet assays were employed to assess DNA damage levels. Consistent with our findings, DNA damage was observed when SK-MES1 and H2030 cells treated with etoposide and comet assays revealed that ARID1B knockdown in SK-MES1 and H2030 cells resulted in an increased tail length, indicating enhanced DNA damage upon etoposide treatment (Fig. 3C). The statistical analysis of our Fig. 3C results demonstrated that in both the H2030 and SK-MES1 cell lines, the longest comet tail length was observed when ARID1B was knocked down and the cells were treated with etoposide. The next longest comet tails were observed when normal cells were treated with etoposide. However, when ARID1B was solely knocked down without etoposide treatment, the changes were not significant.

### ARID1B is related to cell chromatin accessibility

The DNA damage response (DDR) occurs in the context of chromatin, and architectural features of chromatin have played significant role in DNA damage signaling and repair. DNA break induced chromatin remodeling affects repair factor access and choice. Chromatin relaxation followed by condensation represent the initial effects of DSBs, which are necessary for both DSB damage and repair DNA damage accumulation may be impacted by altered chromatin and chromosome structures. Since SWI/SNF chromatin remodeling complexes uses the energy of ATP to change the structure of DNA, playing key roles in DNA regulation and repair. To determine whether ARID1B is responsible for the abnormal compaction of chromatin structures in associated DNA damage, micrococcal nuclease digestion assay was then carried out on both cell lines to determine whether ARID1B expression would alter the chromatin conformation. Larger diffuse DNA fragments can be seen in the chromatin of ARID1B knocking down cells following the addition of MNase and the DNA fragment is more degraded than the control group, which indicated that the chromatin structure is looser in ARID1B knockdown cells. These results demonstrate that reduced ARID1B gene expression increased the accumulation of DNA damage during DNA damage and exhibited increased chromatin accessibility (Fig. 3D, E). We conducted ATAC-seq analysis on SK-MES-1 cells and SK-MES-1 cells with shRNA-mediated knockdown of ARID1B, revealing the distribution of peaks obtained on the chromosomes. The chromatin accessibility of ARID1B-knockdown SK-MES-1 cells was found to be increased, indicating a higher accessibility of chromatin in these cells (Fig. 3F).

Collectively, these findings provide further evidence that ARID1B knockdown enhances DNA damage in SK-MES1 and H2030 cells, as demonstrated by increased γH2AX levels, prolonged comet tail length, and enhanced chromatin accessibility. These results suggest a crucial role for ARID1B in maintaining DNA integrity and highlight its involvement in DNA damage response pathways.

### ARID1B Expression Correlates with Immune Suppression in NSCLC

**Figure 4:**
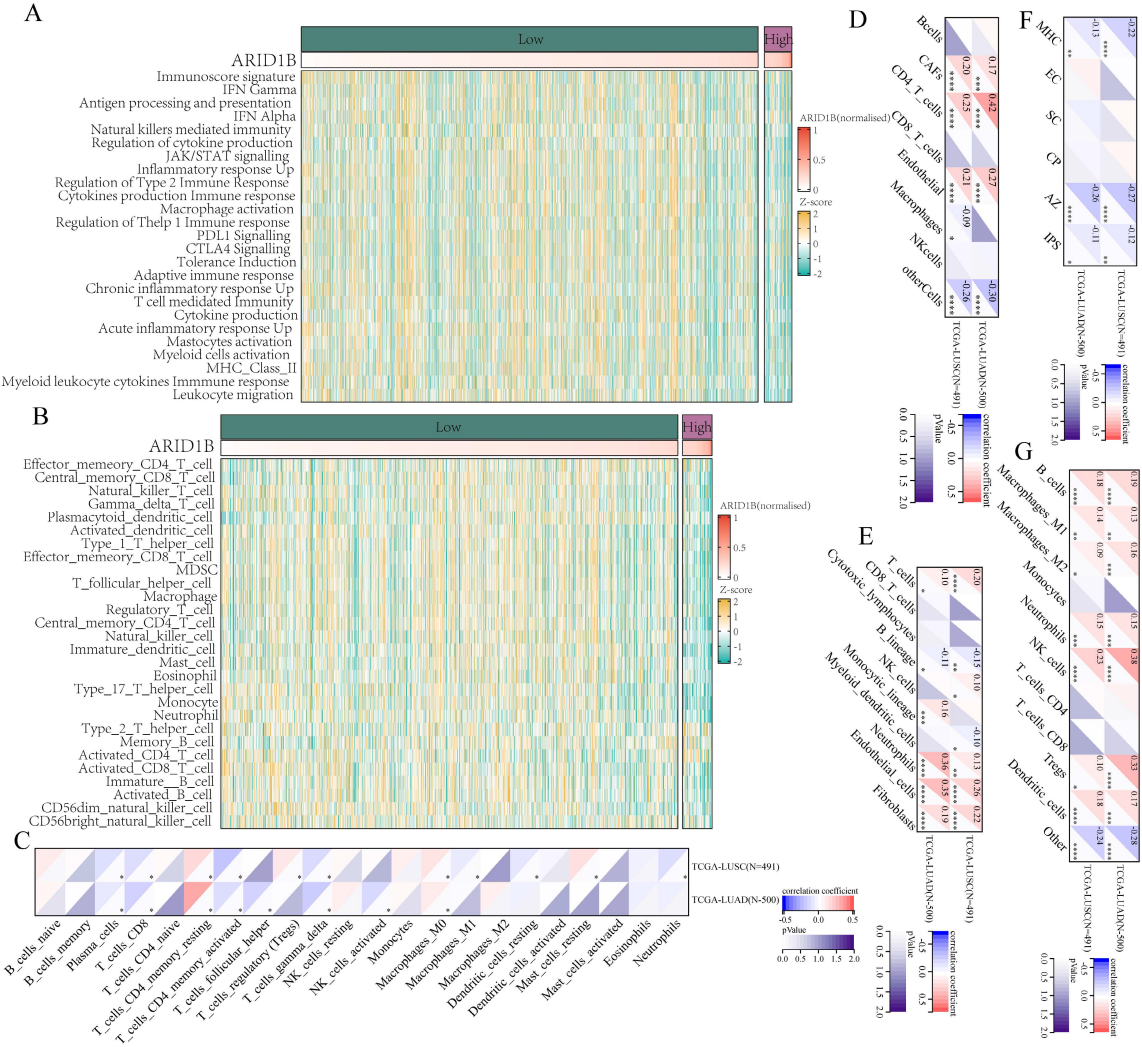
** Association between ARID1B expression and immune microenvironment in NSCLC.** (**A**) Immune scores in NSCLC samples with high or low ARID1B expression based on gene set variation analysis (GSVA). (**B**) Immune cell infiltration levels in NSCLC samples with high or low ARID1B expression based on single-sample gene set enrichment analysis (ssGSEA). (**C-G**) Correlation analysis between ARID1B expression and immune infiltration using CIBERSORT, EPIC, MCPcounter, IPS, and QUANTISEQ methods. *p < 0.05, **p < 0.01, ***p < 0.001, ****p < 0.0001.

To determine whether ARID1B deficiency alter immune microenvironment, we collected data from TCGA and analyzed 25 immune-related pathways. Using gene set variation analysis (GSVA), we scored each tumor sample to represent its immune microenvironment. Based on ARID1B gene expression levels, we divided the samples into high and low expression groups, using a TPM value of 30 as the cutoff, with values above 30 indicating high expression and values below indicating low expression. Our analysis revealed that the ARID1B high-expression group exhibited a significantly lower immune score compared to the ARID1B low-expression group, indicating a state of immune suppression (Fig. 4A).

To validate this observation, we utilized the single-sample gene set enrichment analysis (ssGSEA) method to assess tumor immune cell infiltration. Consistently, we found that patients with high ARID1B expression showed reduced levels of immune cell infiltration compared to the low-expression group (Fig. 4B).

Furthermore, we employed five additional methods, including CIBERSORT (Fig. 4C), EPIC (Fig. 4D), MCPcounter (Fig. 4E), IPS (Fig. 4F), and QUANTISEQ (Fig. 4G), to evaluate immune infiltration in the tumor microenvironment. Correlation analysis between their results and ARID1B expression revealed a negative correlation between ARID1B expression and the majority of immune-related cells or functions. In our CIBERSORT analysis, we observed a negative correlation between the infiltration of CD8+ T cells in NSCLC and the expression of ARID1B (Fig. 4C). The results of the other four immune infiltration methods were consistent with CIBERSORT, but did not reach statistical significance. The EPIC analysis (Fig. 4D) and MCPcounter analysis (Fig. 4E) revealed a positive correlation between ARID1B and tumor-associated fibroblasts. Similarly, the IPS analysis (Fig. 4F) showed a negative correlation between ARID1B expression and tumor immunogenicity.

Taken together, our findings consistently demonstrate that high expression of ARID1B in NSCLC is associated with immune suppression, as evidenced by lower immune scores, reduced immune cell infiltration, and negative correlations with multiple immune-related cell types and functions. These results suggest that ARID1B may play a critical role in modulating the immune landscape of NSCLC tumors.

### ARID1B regulates the immune microenvironment through cGAS-STING pathway

**Figure 5:**
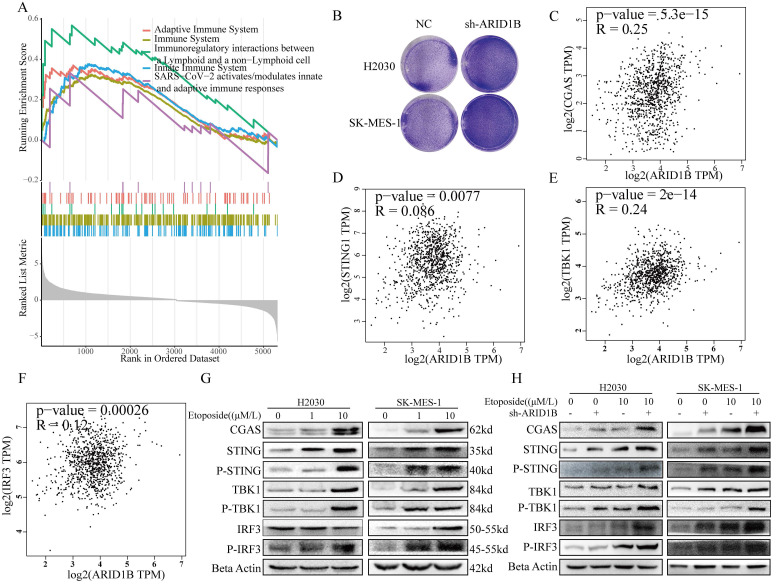
** ARID1B modulation of the cGAS-STING pathway.** (**A**) Gene set enrichment analysis (GSEA) showing changes in immune-related pathways following ARID1B mutation. B Before and after knocking down ARID1B, co-culture it with T cells to detect its degree of T cell killing. (**C-F**) Correlation analysis between ARID1B expression and genes related to the cGAS-STING pathway (CGAS, STING1, TBK1, and IRF3) in TCGA datasets. (**G**) Western blot demonstrating the activation of the cGAS-STING pathway upon cellular damage induction. (**H**) Western blot analysis showing enhanced cGAS-STING pathway activation in ARID1B knockdown lung cancer cells following cellular damage induction.

The gene set enrichment analysis (GSEA) of differentially expressed genes revealed significant changes in multiple immune-related pathways following ARID1B mutation (Fig. 5A). Knocking out ARID1B enhanced the susceptibility of two subtypes of non-small cell lung cancer cells to T cell-mediated cytotoxicity (Fig. 5B), and knocking out ARID1B increased the number of lung cancer cells dying when co cultured with T cells in both subtypes of non-small cell lung cancer. We investigated the apoptotic response of arid1b-knockdown non-small cell lung cancer (NSCLC) cells, and the results indicated that the reduction of arid1b did not influence the cellular apoptosis (Supplementary Fig. 4A,B). This substantiates that the observed apoptosis, occurring in co-culture with T cells, is mediated by T cells.

Furthermore, we investigated the relationship between ARID1B and the cGAS-STING pathway. We found that ARID1B positively correlated with CGAS (Fig. 5C), TBK1 (Fig. 5E), and IRF3 (Fig. 5F), but showed no association with STING1(Fig. 5D) expression.

To validate these findings, we performed Western blot analysis. We observed that induction of cellular damage led to an upregulation of the cGAS-STING pathway (Fig. 5G). Interestingly, cells with ARID1B knockdown showed enhanced upregulation of the cGAS-STING pathway upon damage induction (Fig. 5H).

Collectively, our results demonstrate that ARID1B mutation alters immune-related pathways, as evidenced by significant changes in their enrichment. Additionally, we identified associations between ARID1B and key components of the cGAS-STING pathway. The induction of cellular damage resulted in the upregulation of the cGAS-STING pathway, and cells with ARID1B knockdown exhibited an augmented response in the pathway upon damage induction. These findings suggest that ARID1B plays a role in modulating the cGAS-STING pathway in NSCLC.

## Discussion

The SWI/SNF complex is a critical regulator of chromatin structure and gene expression, and its dysfunction has been implicated in various cancers. In our study, we focused on the impact of ARID1B mutation, a core member of the SWI/SNF complex, on the response to ICIs in lung cancer patients. Our findings shed light on the potential mechanisms underlying the favorable response observed in SWI/SNF-mutated patients and provide important implications for precision medicine and immunotherapy.

SWI/SNF complexes play an important role in the maintenance of genome integrity and their role in DDR includes modification of chromatin structure around sites of DNA damage and direct recruitment of proteins required for DDR 7. During DNA damage, SWI/SNF complexes rapidly bind to chromatin surrounding DNA damage through interaction with γ-H2AX 24. Canonical BAF and PBAF complexes have been shown to be involved in both nonhomologous end joining and homologous recombination repair processes 7.

ARID1 proteins recruit the BAF complexes to DNA damage sites, assist in homologous recombination-mediated DNA repair and nonhomologous end joining at double-strand breaks, and required for cellular resistance to various types of DNA damage 7,25. It was shown that ARID1A regulates DNA double-strand break repair through interaction with DNA damage checkpoint kinases ATR and ATM. Moreover, ARID1A interacts with TOP2a that resolves newly replicated sister chromatids linked by catenated strands of DNA and prevents DNA entanglements during mitosis. In addition, ARID1A induces expression of a subunit of the cohesion complex STAG1, which plays an important role in telomere cohesion and stabilization 7. Inactivation of ARID1A results in mitotic defects such as anaphase bridges and chromosomal lagging, leading to genomic instability and polyploidy. ARID1A was also found to interact with the mismatch repair (MMR) protein MSH2 and recruit it to chromatin during DNA replication to induce MMR 26. However, the mechanisms about ARID1B with DDR and ICIs therapy are not well understood.

Here, we observed a significant association between ARID1B mutation and improved overall survival in lung cancer patients treated with ICIs, consistent with previous studies 8. This highlights the potential of ARID1B mutation as a predictive biomarker for immunotherapy outcomes in non-small cell lung cancer (NSCLC) patients. Our study further adds to the growing body of evidence supporting the role of SWI/SNF complex mutations as potential biomarkers for immune checkpoint blockade.

In our investigation of the functional consequences of ARID1B mutation, we explored its impact on DNA damage response and repair mechanisms. ARID1B has been implicated in maintaining genome stability and influencing the cellular response to DNA damage. We found that ARID1B-deficient cells exhibited enhanced DNA damage, as evidenced by increased levels of phosphorylated CHK1, CHK2, ATM, and γH2AX 27-30. These findings suggest that ARID1B mutation may impair DNA damage repair pathways, rendering cancer cells more susceptible to the cytotoxic effects of ICIs. This is consistent with studies implicating SWI/SNF complex members in DNA damage repair and genome integrity maintenance.

**Figure 6:**
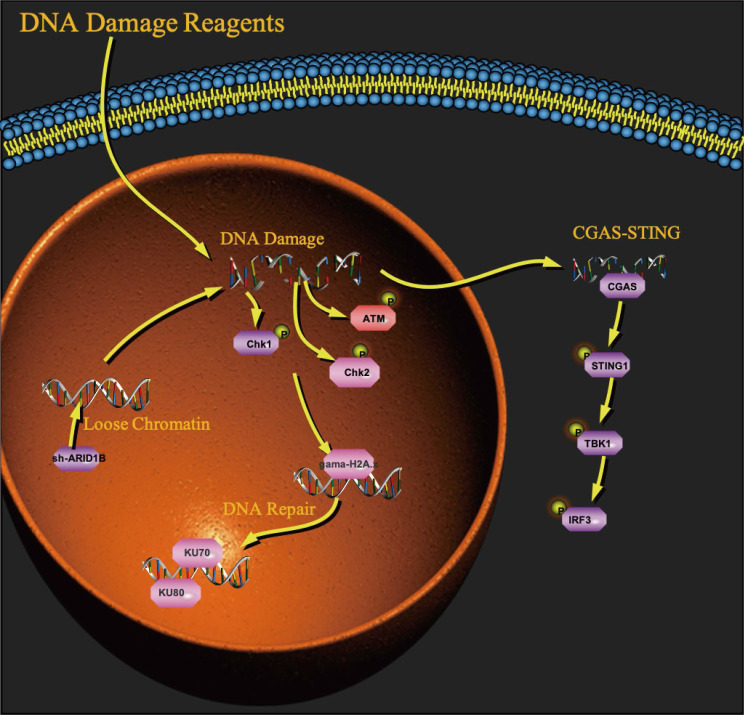
A working model for the role of ARID1B in activating the cGAS-STING pathway.

Furthermore, our study revealed an additional mechanism by which ARID1B deficiency can influence the tumor microenvironment and enhance the response to immunotherapy. Our research indicates that the absence of ARID1B enhances chromatin accessibility in cells, which increases their likelihood of DNA damage. As a receptor for cytoplasmic DNA fragments, the Sting pathway's association with the ARID1B gene is undoubtedly our next research direction. We found that ARID1B-deficient cells exhibited activation of the cGAS-STING signaling pathway (Fig. 6) 31-39, which is involved in the innate immune response to DNA damage and viral infection. Activation of this pathway can lead to the production of type I interferons and pro-inflammatory cytokines, promoting an immune-activated tumor microenvironment. This activation of the immune response could potentially enhance the cytotoxic function of T cells and improve the anti-tumor immune response.

The significance of our study lies in several aspects. Firstly, we provide further evidence supporting the association between ARID1B mutation and improved response to ICIs in NSCLC patients. This strengthens the potential of ARID1B mutation as a predictive biomarker for immunotherapy outcomes. Secondly, our study unravels the functional implications of ARID1B mutation in DNA damage response and repair, providing mechanistic insights into the underlying biology. Moreover, we highlight the cGAS-STING signaling pathway as a potential mediator of the immune-activated tumor microenvironment in ARID1B-deficient cells, further enhancing our understanding of the complex interplay between genomic alterations and the immune system in cancer.

In conclusion, our study expands the understanding of the mechanisms by which ARID1B mutation influences the response to ICIs in lung cancer patients. ARID1B mutation may impair DNA damage repair pathways and activate immune signaling pathways, leading to improved sensitivity to immunotherapy. These findings have important implications for precision medicine approaches and the development of targeted therapies aimed at SWI/SNF complex members, such as ARID1B, in lung cancer. Further validation studies in larger patient cohorts and functional investigations are warranted to fully elucidate the clinical relevance and therapeutic implications of these findings.

### Limitations

Our study lacks in vivo models constructed by ARID1B mutant and wild-type, and cannot directly verify the direct response of ARID1B mutant and wild-type to ICI treatment.

## Supplementary Material

Supplementary figures.
