# Supplementary material for: ARID1B Deficiency Leads to Impaired DNA Damage Response and Activated cGAS-STING Pathway in Non-Small Cell Lung Cancer
Source: J Cancer. 2024 Mar 11;15(9):2601–12. doi: 10.7150/jca.91955 (PMC10988295; doi:10.7150/jca.91955)
Supplement: Supplementary file 1 — Supplementary figures. [file jcav15p2601s1.pdf]

Supplementary Figure 1: Mutation frequency and types， as well as predicted PTM sites of ARID1B in NSCLC patients from AACR GENIE project. The distribution of mutation types across the exons of ARID1B is shown.

Supplementary Figure 2: **The effect of ARID1B mutation on signaling pathways in NSCLC. A** GO Pathway enrichment analysis of differentially expressed genes between ARID1B-mutated and non-mutated NSCLC patients. The bar graph represents the significant enrichment in pathways related to chromatin, DNA assembly and stability, and innate immunity. **B** Gene set enrichment analysis (GSEA) revealing downregulation of chromatin and DNA assembly/stability-related pathways in ARID1B-mutated NSCLC patients.

Supplementary Figure 3: **Correlation analysis between ARID1B gene expression and DNA damage response and repair related genes in NSCLC.** (**A-F**) Correlation analysis between the expression of DNA damage response and repair genes (ATM, CHEK1, CHEK2, H2AFX, KU70, and KU80) and the expression of ARID1B in TCGA datasets.

Supplementary Figure 4: The results of cell apoptosis in non-small cell lung cancer cells H2030(A) and SK-MES-1(B) after ARID1B knockdown.

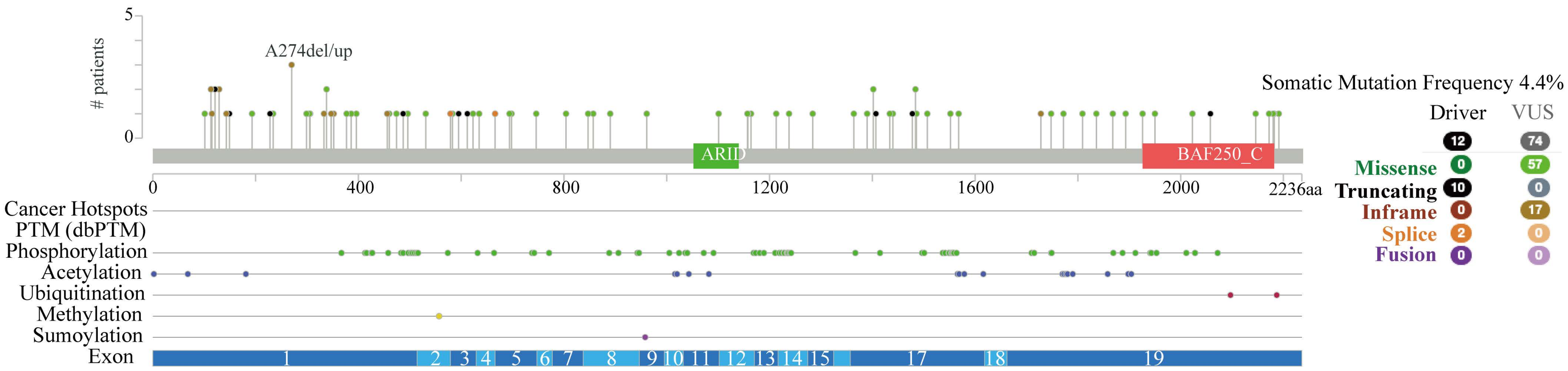
5
0
patients
A274del/up
ARID
BAF250_C
0
400
800
1200
1600
2000
2236aa
Cancer Hotspots
PTM (dbPTM)
Phosphorylation
Acetylation
Ubiquitination
Methylation
Sumoylation
Exon
1 2 3 4 5 6 7 8 9 10 11 12 13 14 15 17 18 19
Somatic Mutation Frequency 4.4%
Driver
VUS
12
74
Missense 0 57
Truncating 10 0
Inframe 0 17
Splice 2 0
Fusion 0 0

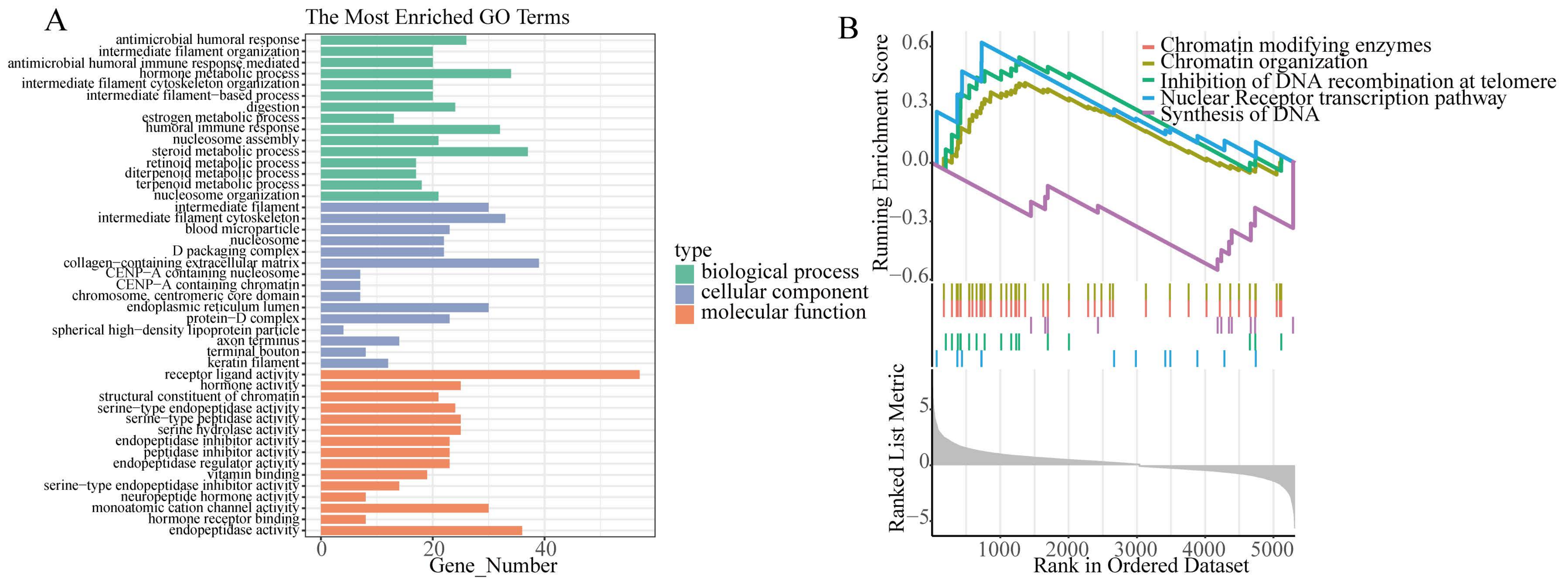
A
The Most Enriched GO Terms
antimicrobial humoral response
intermediate filament organization
antimicrobial humoral immune response mediated
hormone metabolic process
intermediate filament cytoskeleton organization
intermediate filament−based process
digestion
estrogen metabolic process
humoral immune response
nucleosome assembly
steroid metabolic process
retinoid metabolic process
diterpenoid metabolic process
terpenoid metabolic process
nucleosome organization
intermediate filament
intermediate filament cytoskeleton
blood microparticle
nucleosome
D packaging complex
collagen−containing extracellular matrix
CENP−A containing nucleosome
CENP−A containing chromatin
chromosome, centromeric core domain
endoplasmic reticulum lumen
protein−D complex
spherical high−density lipoprotein particle
axon terminus
terminal bouton
keratin filament
receptor ligand activity
hormone activity
structural constituent of chromatin
serine−type endopeptidase activity
serine−type peptidase activity
serine hydrolase activity
endopeptidase inhibitor activity
peptidase inhibitor activity
endopeptidase regulator activity
vitamin binding
serine−type endopeptidase inhibitor activity
neuropeptide hormone activity
monoatomic cation channel activity
hormone receptor binding
endopeptidase activity
0
20
40
Gene_Number
type
biological process
cellular component
molecular function
B
Running Enrichment Score
0.6
0.3
0.0
−0.3
−0.6
Chromatin modifying enzymes
Chromatin organization
Inhibition of DNA recombination at telomere
Nuclear Receptor transcription pathway
Synthesis of DNA
Ranked List Metric
5
0
−5
1000
2000
3000
4000
5000
Rank in Ordered Dataset

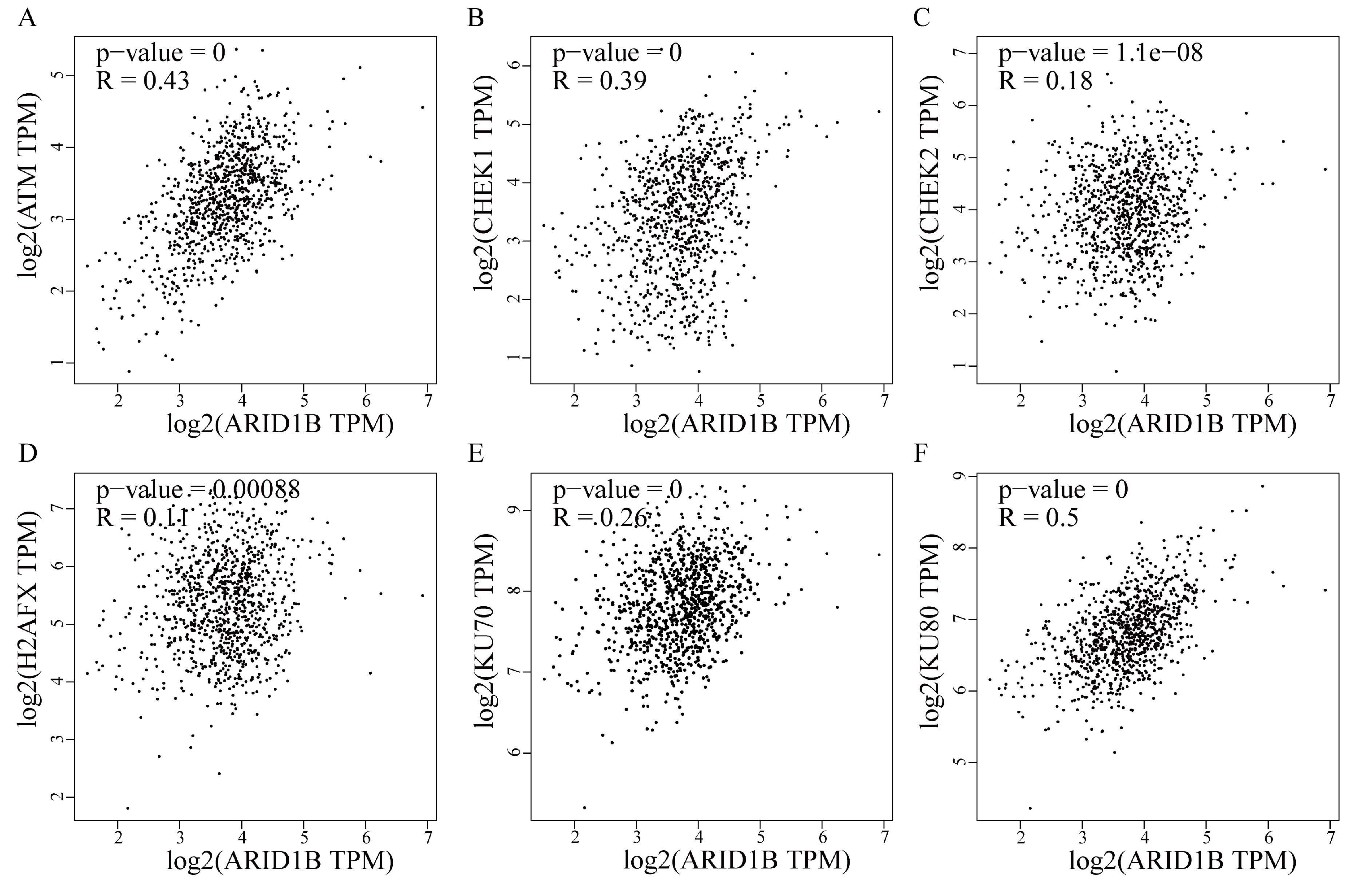
A
p−value = 0
R = 0.43
log2(ATM TPM)
log2(ARID1B TPM)
B
p−value = 0
R = 0.39
log2(CHEK1 TPM)
log2(ARID1B TPM)
C
p−value = 1.1e−08
R = 0.18
log2(CHEK2 TPM)
log2(ARID1B TPM)
D
p−value = 0.00088
R = 0.11
log2(H2AFX TPM)
log2(ARID1B TPM)
E
p−value = 0
R = 0.26
log2(KU70 TPM)
log2(ARID1B TPM)
F
p−value = 0
R = 0.5
log2(KU80 TPM)
log2(ARID1B TPM)

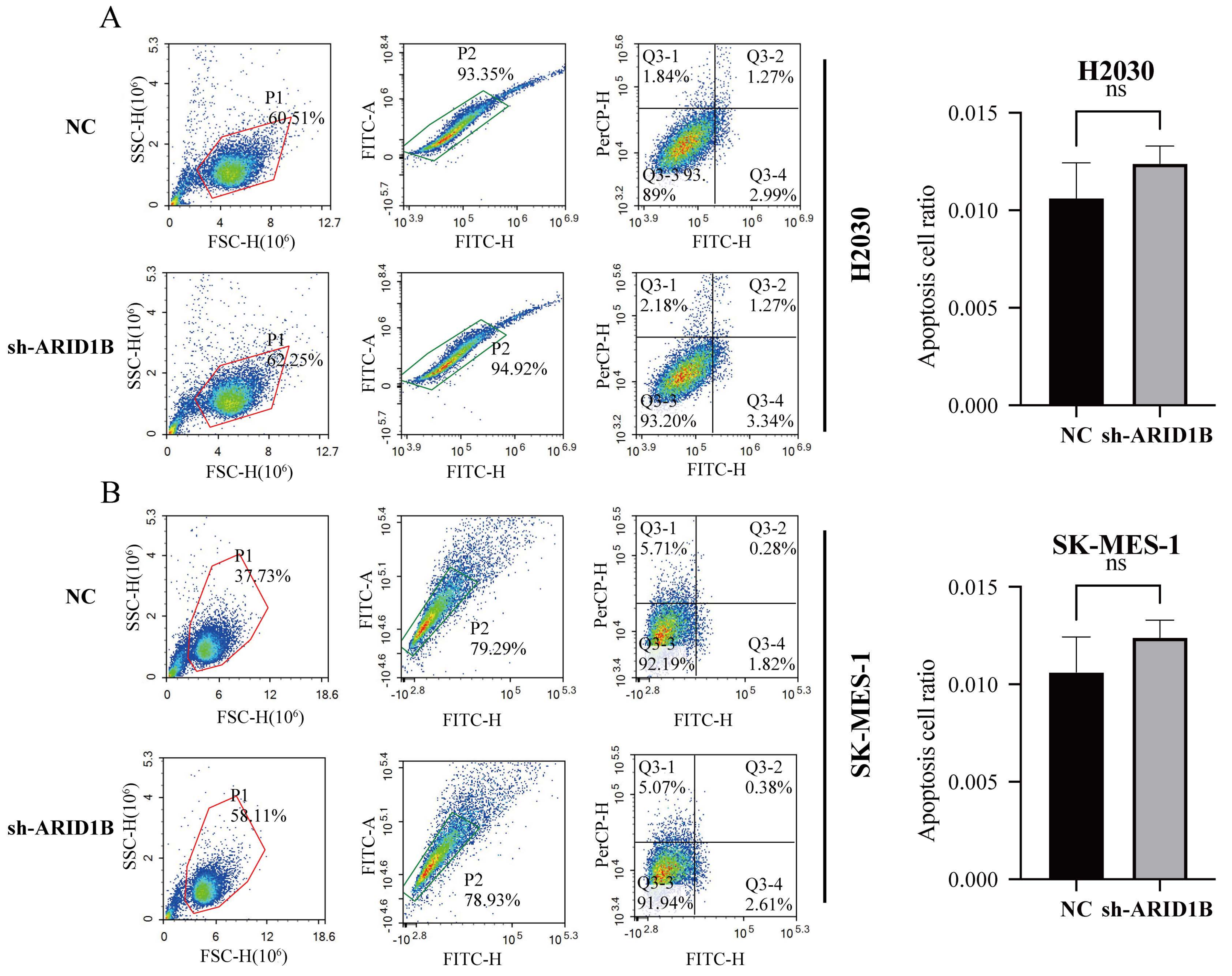

A
NC
sh-ARID1B
P1
60.51%
62.25%
P2
93.35%
94.92%
Q3-1
1.84%
2.18%
Q3-2
1.27%
Q3-3 93.
89%
93.20%
Q3-4
2.99%
3.34%
SSC-H(10⁶)
FSC-H(10⁶)
FITC-A
FITC-H
PerCP-H
H2030
ns
Apoptosis cell ratio
B
37.73%
58.11%
79.29%
78.93%
5.71%
0.28%
92.19%
1.82%
5.07%
0.38%
91.94%
2.61%
SK-MES-1
